# Analysis of Cannabinoid-Containing Fluids in Illicit Vaping Cartridges Recovered from Pulmonary Injury Patients: Identification of Vitamin E Acetate as a Major Diluent

**DOI:** 10.3390/toxics8010008

**Published:** 2020-01-24

**Authors:** Bryan Duffy, Lingyun Li, Shijun Lu, Lorie Durocher, Mark Dittmar, Emily Delaney-Baldwin, Deepika Panawennage, David LeMaster, Kristen Navarette, David Spink

**Affiliations:** 1 Laboratory of Organic Analytical Chemistry, Wadsworth Center, New York State Department of Health, Albany, NY 12201, USA; bryan.duffy@health.ny.gov (B.D.); lingyun.li@health.ny.gov (L.L.); shijun.lu@health.ny.gov (S.L.); lorie.durocher@health.ny.gov (L.D.); mark.dittmar@health.ny.gov (M.D.); emily.delaney-baldwin@health.ny.gov (E.D.-B.); deepika.panawennage@health.ny.gov (D.P.); 2 Department of Environmental Health Sciences, School of Public Health, University at Albany, State University of New York, Rensselaer, NY 12144, USA; 3 Laboratory of Molecular Diagnostics, Wadsworth Center, New York State Department of Health, Albany, NY 12201, USA; david.lemaster@health.ny.gov; 4 Center for Environmental Health, New York State Department of Health, Albany, NY 12201, USA; kristen.navarette@health.ny.gov; 5 Albany Medical Center, Department of Pediatrics, Albany, NY 12208, USA

**Keywords:** electronic cigarettes, vaping cartridges, cannabinoids, vitamin E acetate

## Abstract

Beginning in June of 2019, there was a marked increase in reported cases of serious pulmonary injury associated with vaping. The condition, referred to as e-cigarette or vaping product use-associated lung injury (EVALI), does not appear to involve an infectious agent; rather, a chemical adulterant or contaminant in vaping fluids is suspected. In August of 2019, the Wadsworth Center began receiving vaporizer cartridges recovered from patients with EVALI for analysis. Having no a priori information of what might be in the cartridges, we employed untargeted analyses using gas chromatography-mass spectrometry and high-resolution mass spectrometry to identify components of concern. Additionally, we employed targeted analyses used for New York medical marijuana products. Here, we report on the analyses of 38 samples from the first 10 New York cases of EVALI for which we obtained cartridges. The illicit fluids had relatively low cannabinoid content, sometimes with unusual Δ^9^-/Δ^8^-tetrahydrocannabinol ratios, sometimes containing pesticides and many containing diluents. A notable diluent was α-tocopheryl acetate (vitamin E acetate; VEA), which was found in 64% of the cannabinoid-containing fluids. To investigate potential sources of the VEA, we analyzed six commercial cannabis-oil diluents/thickeners. Three were found to be >95% VEA, two were found to be primarily squalane, and one was primarily α-bisabolol. The cause(s) of EVALI is unknown. VEA and squalane are components of some personal care products; however, there is growing concern that vaping large amounts of these compounds is not safe.

## 1. Introduction

Inhaling aerosolized liquids, or vaping, has become an increasingly popular method for drug delivery. Vaping of nicotine products has been touted as a safer alternative to smoking, particularly among those undergoing cigarette smoking-cessation. Vaping is also a popular method for the delivery of cannabinoids in states that permit adult use of marijuana products and/or have state-sanctioned medical marijuana programs. In addition to legal cannabis product sales in many states, there remain black markets for cannabis products, of which the sale of illicit vaporizer cartridges constitutes a major proportion. The safety of these black-market cannabis vaporizer cartridges and the contents within them regarding contamination and/or adulteration remains a major concern.

Beginning in June of 2019, there was a sudden increase in reported cases of serious pulmonary injury that appeared to be associated with vaping in the states of Illinois and Wisconsin in the U.S. [1]. The condition [2], now referred to as e-cigarette or vaping product use-associated lung injury (EVALI), did not appear to involve an infectious agent; rather, a chemical adulterant or contaminant in the vaping cartridges was suspected. It soon became apparent that the outbreak of vaping-associated pulmonary injury was not confined to Illinois and Wisconsin, as cases were soon being documented in over 30 states in the U.S. [2,3]. Officials of the New York State Department of Health (NYSDOH) have received reports of cases occurring in all regions of New York State (NYS). In August of 2019, the Wadsworth Center of the NYSDOH, through its associations and support of the Poison Control Centers in NYS, received requests for analysis of vaporizer cartridges that were recovered from patients who had been hospitalized with the pulmonary syndrome. As additional reports of vaping-associated pulmonary injury were received in August of 2019, NYSDOH launched an investigation. The Center for Environmental Health (CEH) and the Wadsworth Center began working in concert to characterize the disease outbreak in NYS and to investigate the possible causes. The role of the Wadsworth Center was to analyze case-associated vaporizer cartridges for possible contamination and/or adulteration that may be related to the pulmonary condition.

Having no a priori information or prediction of what contaminants or adulterants might be present in the fluids, we employed untargeted analyses in the same manner that we use in identifying unknown and suspicious materials, such as white powders and suspected drugs and toxicants submitted through Poison Control Centers in NYS and the NYS Police. We performed a series of untargeted analyses of the vaporizer fluids employing gas chromatography-mass spectrometry (GC-MS) and liquid chromatography-high resolution tandem mass spectrometry (LC-HRMS/MS) to detect and identify potential additives, contaminants and organic toxicants. We also used methods employing high-performance liquid chromatography with photodiode array detection (HPLC-PDA) and liquid chromatography-tandem mass spectrometry (LC-MS/MS) with multiple reaction monitoring (MRM) that are routinely performed in our NYSDOH Medical Marijuana Laboratory to assess the quality of medical marijuana products produced in NYS. Here, we report on the comprehensive analysis of semi-volatile organic chemicals in 38 samples from the first 10 cases of EVALI in NYS for which we obtained vaporizer fluids. Our results revealed numerous analytical findings, the most notable being the identification of α-tocopheryl acetate (vitamin E acetate, or VEA) as a major diluent in illicit cannabis vaporizer cartridges recovered from EVALI patients.

## 2. Materials and Methods

### 2.1. Sample Procurement

CEH and the Wadsworth Center worked closely with Poison Control Centers and health care providers in NYS to procure case-associated vaporizer cartridges and devices for analysis. CEH medical staff reviewed medical records for each case reported to NYSDOH to confirm that each was consistent with the clinical description of EVALI provided by the U.S. Centers for Disease Control and Prevention (CDC). When case-associated vaporizer cartridges and devices were available, arrangements were made for these samples to be submitted to the Wadsworth Center for analysis. Upon receipt at Wadsworth, samples were examined, photographed, and any packing and/or markings on the cartridges were noted. Each sample was entered into the Wadsworth Center Clinical Laboratory Information and Management System together with relevant information provided by the submitter.

### 2.2. Sample Processing

Aliquots of residual vaporizer fluid were carefully removed and accurately weighed. Vaporizer cartridges containing only small amounts of fluid were processed by inverting the cartridge in a 15-mL centrifuge tube and centrifuging at 2000× *g* for 1 min to recover the fluid. For analysis, approximately 10 mg of residual fluid, if available, was accurately weighed (to ±0.01 mg) into a new 2-mL polypropylene centrifuge tube and diluted with GC/MS-grade acetonitrile (1.00 mL) to give a suitable stock solution for analysis on several instrumental platforms. When less than 10 mg of fluid was available, the amount of acetonitrile added was adjusted accordingly to maintain a similar concentration.

### 2.3. Analytical Standards and Reagents

Certified cannabinoid reference standards, including cannabidiolic acid (CBDA), cannabigerolic acid (CBGA), cannabigerol (CBG), cannabidiol (CBD), tetrahydrocannabivarin (THCV), cannabinol (CBN), Δ^9^-tetrahydrocannabinol (Δ^9^-THC), Δ^8^-tetrahydrocannabinol (Δ^8^-THC), cannabichromene (CBC), cannabidivarin (CBDV), and Δ^9^-tetrahydrocannabinolic acid-A (THCA) were purchased from Cerilliant (Round Rock, TX, USA). Primary analytical standards and ^13^C-isotopically labeled internal standards for aflatoxins B1, B2, G1, G2, and ochratoxin A were obtained from Romer Labs, (Union, MO, USA). VEA (α-tocopheryl acetate) and vitamin E-*d*_6_ ((±)-α-tocopherol-*d*_6_) standards were purchased from Cerilliant (Round Rock, TX, USA). The myclobutanil standard was purchased from Accustandards (New Haven, CT, USA). Squalane, α-bisobolol, triethyl citrate, glyceryl trioctanoate, glyceryl tridecanoate, isophytol, norgestrel, and myclobutanil-(phenyl-*d*_4_) were purchased from MilliporeSigma (St. Louis, MO, USA). The piperonyl butoxide (PBO) standard was from Agilent Technologies (Santa Clara, CA, USA). Piperonyl butoxide-*d*_9_ (PBO-*d*_9_) was obtained from Toronto Research Chemicals, Toronto, Canada. Medium-chain triglyceride (MCT) oil, Miglyol, was obtained from Warner Graham (Cockeysville, MD, USA). Synthetic cannabinoid standards, including AB-FUBINACA, XLR11, AB-PINACA, AB-CHMINACA, MAB-CHMINIACA, FUB-PB-22, MDMB-FUBINACA, NM2201, ADB- FUBINACA MMB-FUBINACA and CB-13, and the fentanyl analog screening kit, with 150 synthetic opioid compounds, including 140 fentanyl analogs, were obtained from Cayman Chemical (Ann Arbor, MI, USA). For use in our pesticide screening method, mixtures containing 884 pesticide and pesticide metabolite standards [4] were kindly provided by Dr. Jon Wong of the Center for Food Safety and Applied Nutrition, US Food and Drug Administration (FDA), College Park, MD, USA. Ammonium formate, formic acid, methanol, acetonitrile, and water were HPLC-grade. All other reagents used were analytical grade.

### 2.4. GC-MS Untargeted Analysis

The initial vaporizer fluid solutions at approximately 10 mg/mL were used for full-scan GC-MS analysis. Analyses were conducted using a 6890A gas chromatograph interfaced with a 5973N quadrupole Mass Selective Detector (MSD; Agilent Technologies). The injection volume was 5 μL, and analyte separation was accomplished using an Agilent J&W DB5-MS capillary column (60 m × 250 μm ID with 0.25 μm film thickness) using ultra-high-purity helium as the carrier gas at a constant flow rate of 1.5 mL/min (Airgas, Radnor, PA, USA). The MS transfer line and ion source were maintained at 300 °C and 235 °C, respectively. The solvent delay was 7 min. Data acquisition was in the full-scan mode over the range of 50 to 550 *m/z*. To provide for maximal resolution of components in complex mixtures and to allow for library-searchable mass spectra of the resolved components to be recorded, a GC temperature program with a very slow rate of increase was employed. Initially, the oven was at 90 °C for 1 min, which was followed by a ramp of 2 °C/min to 320 °C; followed by a hold at 320 °C for 25 min. MSD Enhanced ChemStation version E.02.01 (Agilent Technologies) was used for system control and data processing. MS Search 2.0 software and the National Institute of Science and Technology (NIST) Mass Spectral Library 11 were used for compound identification. The most recent Cayman toxicology mass spectral library was also queried for possible controlled substances and the presence of synthetic cannabinoids and opioids.

### 2.5. LC-HRMS/MS Untargeted Analysis

Untargeted LC-HRMS/MS analysis was performed using a Shimadzu HPLC consisting of an SIL-20ACxR autosampler, FCV-11A2 solvent selector and LC-20ADxR pumps interfaced with a high-resolution quadrupole-time-of-flight (TOF) mass spectrometer (TripleTOF MS 6600 system with a DuoSprayTM ion source; SCIEX, Ontario, Canada). The system operated in the positive-ion electrospray ionization (ESI) mode and was calibrated using the external standard calibrant delivery system. A Poroshell EC-C18 HPLC column (Agilent, 2.1 × 100 mm; 2.7-µm particle size) was used. Prior to analysis, the original vaporizer fluid solutions at 10 mg/mL were diluted an additional 500-fold, and 2-µL portions of the diluted samples were injected onto the LC-HRMS/MS system. For gradient elution from the HPLC, mobile phase A was 0.1% *v*/*v* formic acid in water and mobile phase B was 5 mM ammonium formate in methanol. The flow rate was 0.250 mL/min, and the column oven was maintained at 40.0 °C. The binary gradient was initially at 20% B and increased to 100% B over 15 min. After a hold at 100% B for 5 min, the composition was returned to 20% B in 0.1 min and equilibrated for 5 min prior to the next injection.

The instrument was operated in the positive-ion ESI mode for high-resolution MS and MS/MS acquisition. Information-dependent acquisition was used with a TOF-MS survey scan from 70 to 2000 *m/z*, and five dependent MS/MS scans were recorded using a collision energy of 35 eV with collision energy spread ±15 eV. Data were acquired using Analyst Software (SCIEX, version 1.6.1); data were processed using PeakView software (SCIEX, version 2.1). When the original vaporizer fluid solutions were diluted as described, the synthetic cannabinoids and opioids could be detected at a level of 0.05% by mass in the original vaporizer fluid.

To augment our available commercial and public domain databases, we continually expand our in-house accurate mass compound library by analyzing the increasing number of commercially available analytical standards of synthetic cannabinoids, opiates, synthetic opioids, stimulants, and other drugs of abuse and controlled substances. Molecular formulas, accurate masses, MS/MS spectra and HPLC retention times are included in the library. When a new synthetic cannabinoid or opioid is reported to be in use, the accurate mass of the compound is added to our acquisition target list for potential detection in subsequent analyses and a certified standard, if available, is purchased.

### 2.6. Pesticide Screening Using Orbitrap Mass Spectrometry

We adopted a workflow of non-targeted data acquisition for target analysis using ultra high-performance liquid chromatography (UHPLC) coupled with a quadrupole-orbitrap mass spectrometer (Q-Orbitrap-MS) that is based on previous studies of screening for pesticide residues in various matrices including fruits and vegetables [4,5]. The instrumental system used was a Vanquish UHPLC with a Hypersil GOLD column (100 × 2.1 mm with 1.9-µm particle size, Thermo Fisher Scientific, Waltham, MA, USA) interfaced with a high-resolution Q-Exactive Q-Orbitrap-MS (Thermo Fisher Scientific) operating in the positive-ion ESI mode. The pesticide database was kindly provided by Dr. Jon Wong of the Center for Food Safety and Applied Nutrition, U.S. FDA, College Park, MD, U.S. For positive identification of pesticides in the screening method, the following criteria are established: the UHPLC retention time of the compound must be within 10 s of that of the standard, the accurate mass of the parent ion must be observed within 5 ppm of the theoretical value and the accurate masses of two fragment ions must be observed within 10 ppm of the theoretical values. Exceptions are noted for compounds that do not show a quasi-molecular ion, such as [M+H]^+^ or [M+NH_4_]^+^, or compounds that show only a single fragment ion. For analysis, the original vaporizer fluid solutions at 10 mg/mL in acetonitrile were diluted an additional 10-fold. Since the responses for individual pesticides vary by several hundred-fold or more, we established a conservative cutoff of 1 µg/g in the original (undiluted) vaporizer fluid for positive detection in this screening method Analysis of a 5-µg/L standard mixture at the beginning and the end of each analytical batch verified the continual detection of over 800 pesticides.

### 2.7. Quantitation of Cannabinoids

A quantitative method for the determination of the cannabinoid profiles in medical marijuana products was developed, validated and certified by the New York State Environmental Laboratory Approval Program (ELAP) [6,7]. This HPLC-PDA method has been used since December of 2015 and has proven effective for the analysis of thousands of medical marijuana products. Briefly, a Shimadzu (Kyoto, Japan) HPLC system composed of an SIL-20ACxR autosampler, FCV-11A2 solvent selector, LC-20ADxR pumps and an SPD-M20A PDA detector was used. The method involves resolution of cannabinoids on an Agilent Poroshell 120 column (3.0 × 150 mm with 2.7-µm particle size) using an isocratic elution at 73% *v*/*v* acetonitrile in water with 0.1% *v*/*v* formic acid and quantitation of absorbances at 227 nm relative to that of the norgestrel internal standard [6]. The sample preparation method was slightly modified to use the initial vaporizer fluid solutions above without spiking the surrogate.

### 2.8. Analysis of Mycotoxins

A validated and ELAP-accredited method that was developed in the NYSDOH Medical Marijuana Laboratory for the quantitation of aflatoxins B1, B2, G1, G2, and ochratoxin A in medical marijuana products was used [8]. The method employs LC-MS/MS on a Shimadzu HPLC system consisting of a SIL-20ACxR autosampler, an FCV-11A2 solvent selector and LC-20ADxR pumps interfaced with an AB Sciex 4500 QTRAP mass spectrometer operating in the positive-ion ESI mode. Analytes were detected using scheduled MRM with two MS/MS transitions (quantifier and qualifier) for each analyte and ^13^C-labeled internal standard. The method calls for the extraction of 100 mg of material; however, due to the limited amounts of sample material available in this project, a scaled-down sample extraction method was used to prepare the samples. Details of this analytical method are available [8].

### 2.9. Quantitative Analysis of Myclobutanil and PBO

A method for the analysis of the fungicide myclobutanil and the insecticide synergist PBO was developed, validated and certified in the NYSDOH Medical Marijuana Laboratory [9]. Samples were analyzed using the same LC-MS/MS system as was used for mycotoxin analysis, operating in the positive-ion ESI mode with MRM for specific detection of the analytes, myclobutanil and PBO, and their corresponding internal standards, myclobutanil-(phenyl-*d*_4_) and PBO-*d*_9_. Chromatography of these compounds was performed using an Agilent Poroshell 120 EC-C18, 3.0 × 150 mm column with 2.7-µm particle size using a programed gradient of increasing methanol over a period of 15 min [9]. The *m/z* 289.0→70.0 transition was used for quantitation of myclobutanil, and the m/z 289.0→125.0 transition served as the qualifier. The *m/z* 293.0→70.0 and 293.0→129.0 transitions were used for detection of the myclobutanil-(phenyl-*d*_4_) internal standard. For the analysis of PBO, the analyte transitions were *m/z* 356.2→177.2 and *m/z* 356.2→119.1, and for the PBO-*d*_9_ internal standard, the transitions monitored were *m/z* 365.2→177.2 and *m/z* 365.2→119.1.

### 2.10. Quantitative Analysis of VEA using GC-MS

Quantitative analysis of VEA in vaporizer fluids was performed using GC-MS with electron ionization and operation in the selected-ion monitoring mode. The analytical system used was composed of a model 7890B GC with model G4513A autosampler interfaced with a 5977A MSD (Agilent Technologies). The system was controlled by and data were processed using Mass Hunter Version B07.01 SP/Build 7.1.524.1 software. An Agilent HP-5MS column (30 m × 250 µm with 0.25-µm film thickness) was used. The GC temperature program consisted of an initial temperature of 90 °C for 1 min followed by an increase at 8 °C per min to a final temperature of 290 °C, a hold for 4 min, an increase at 10 °C per min to 300 °C, and a hold for 1 min. Ions monitored for VEA were *m/z* 430 and 165, and *m/z* 436 and 171 were monitored for the vitamin E-*d*_6_ internal standard. Dwell times were 50 ms. A calibration range of 0.039 to 2.5 µg/mL was established with *R*^2^ = 0.9996 for the calibration curve (Figure S1). For analysis, the original vaporizer fluid solutions at 10 mg/mL were diluted an additional 1000- to 10,000-fold, which provided an analytical range of 1 to 100% VEA by mass.

### 2.11. Nuclear Magnetic Resonance (NMR) Spectroscopy

NMR spectroscopy was used to determine the components of commercial cannabis oil diluents and thickeners. All samples were dissolved in CDCl_3_ at an approximate concentration of 10%. After transfer to 5 mm NMR tubes, NMR spectral data were collected on a 600 MHz Bruker Avance III spectrometer equipped with a TXI cryoprobe at 298.1 K. Data were analyzed using the Topspin 3.2.7 software on the spectrometer.

## 3. Results

### 3.1. Untargeted Analysis Identifies VEA 

**Figure 1:**
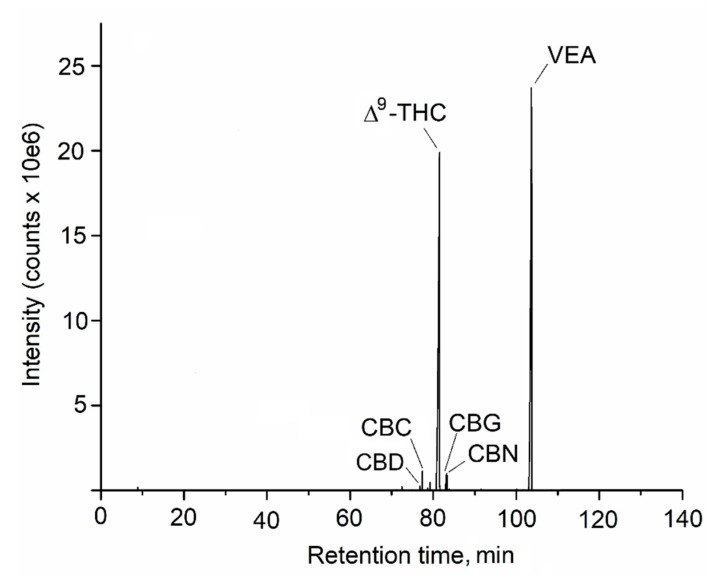
Untargeted analysis of vaporizer fluid using GC-MS identifies VEA. Shown is the TIC chromatogram from the analysis of an EVALI case-associated vaporizer fluid. Identified cannabinoids and VEA are denoted.

When the outbreak of EVALI reached the NYS and we began to receive samples for analysis, we had no preconception of the nature or the number of significant analytes that could be present in these samples. Vaporizer fluids were thus subjected to untargeted analysis using both GC-MS and LC-HRMS/MS platforms. The first results we obtained were notable. Upon analysis of the first cannabis vaporizer oil samples we received using untargeted GC-MS screening, an unknown peak was observed that was the most prominent one in the total-ion current (TIC) chromatogram (Figure 1). 

Upon library search of the National Institute of Standards and Technology (NIST) mass spectral database with the mass spectrum recorded for this component, there was essentially an identical match to the library mass spectrum for α-tocopheryl acetate, or vitamin E acetate (VEA) (Figure S2). When GC-MS analysis of a VEA standard was performed, there were retention time and mass spectral matches for the component in the vaporizer fluid to that of the VEA analytical standard.

**Figure 2:**
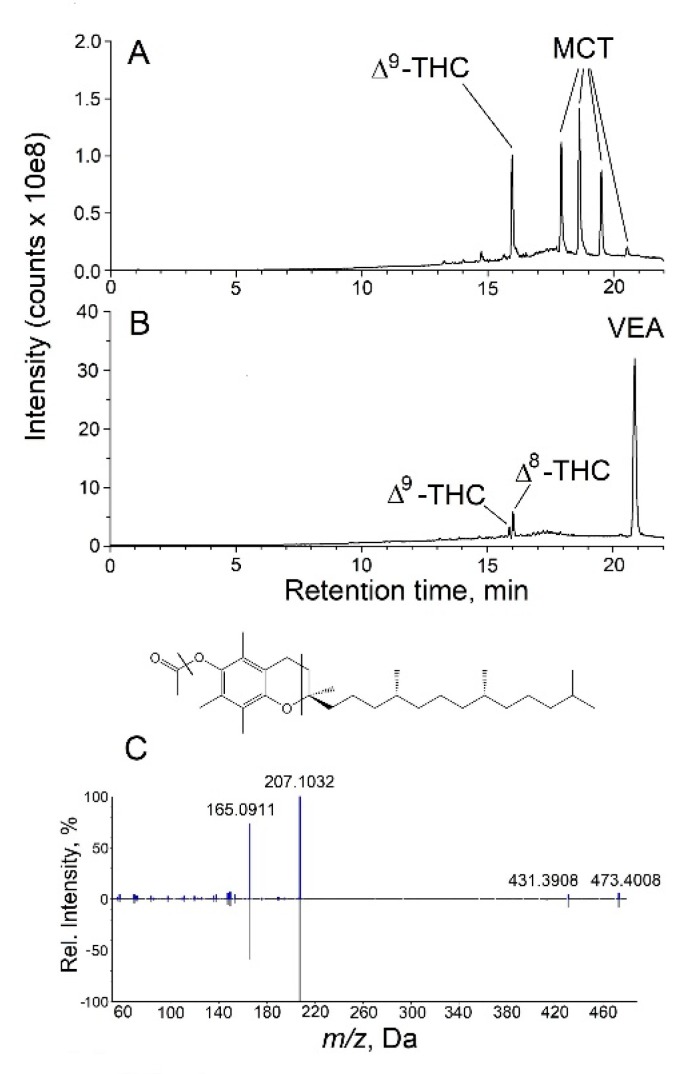
Untargeted analysis of vaporizer fluids using LC-HRMS/MS. Shown in panel (**A**) is the TIC chromatogram from the analysis of a THC-containing vaporizer fluid in which MCT was identified. In panel (**B**), the TIC chromatogram from the analysis of a vaporizer fluid is shown in which Δ^8^-THC, Δ^9^-THC and VEA were identified. In panel (**C**), the high-resolution MS/MS spectrum recorded for VEA in the vaporizer fluid is shown in comparison with that of a VEA standard, with points of fragmentation in the VEA structure as indicated.

Untargeted analysis of vaporizer fluids using LC-HRMS/MS on the Triple-TOF instrument was also performed (Figure 2). While some THC-containing fluids contained MCT as a major diluent (Figure 2A), most of these case-related samples contained VEA (Figure 2B). MCT is a mixture of triglycerides primarily with octanoic acid, decanoic acid or combinations of the two esterified. Based on the high-resolution MS and MS/MS spectra recorded, the peak at retention time 17.92 min from the analysis of a vaporizer fluid represents glyceryl trioctanoate, and the peak at 20.55 min in Figure 2A represents glycerol tridecanoate (Table S1). These assignments were confirmed by analysis of analytical standards (Figure S3). The peak at 18.64 min is consistent with glycerol with two octanoic acid and one decanoic acid moieties esterified, and the peak at 19.51 min with glycerol having one octanoic and two decanoic acid moieties esterified. Accurate mass data supporting these assignments are presented in Table S1. Comparable results were obtained upon analysis of a commercial MCT oil product.

Our untargeted analysis of unknown samples has routinely involved investigation for the presence of synthetic cannabinoids, specifically those of the substituted indole and indazole classes of compounds that have been of recent concern [10,11,12]. While we have detected these compounds in numerous recent analytical studies in support of Poison Control Centers in NYS and the NYS Police, we have not observed any of these compounds in the present study of vaporizer fluids (<0.05% by mass). Likewise, none of the samples we analyzed in this study have shown the presence of opiates or synthetic opioids including fentanyl derivates [13], or compounds in our toxicology screen for controlled substances and drugs of abuse (<0.05% by mass).

As shown in Figure 2B, the major peak in the TIC from the analysis of a different vaporizer fluid represents VEA. The ESI mass spectrum of VEA shows [M + H]^+^, [M + NH_4_]^+^, and [2M + NH_4_]^+^ ions, all of which were confirmed by accurate mass measurements. The high-resolution product-ion spectrum of the [M + H]^+^ ion of VEA at *m/z* 473.4008 (Δ = 4.0 ppm from theoretical) is shown in Figure 2C. The ion at *m/z* 207.1032 is assigned to an ion resulting from cleavage through the dihydropyran ring with charge retained on the substituted dihydroquinone portion of the molecule (Δ = 7.7 ppm from theoretical), and the ion at *m/z* 165.0911 represents a loss of CH_3_CO as well as the dihydropyran ring cleavage (Δ = 0.61 ppm from theoretical). This sample also showed the unusual presence of a significant amount of Δ^8^-THC (Figure 2B).

### 3.2. Targeted Analysis of Vaporizer Fluids

**Figure 3:**
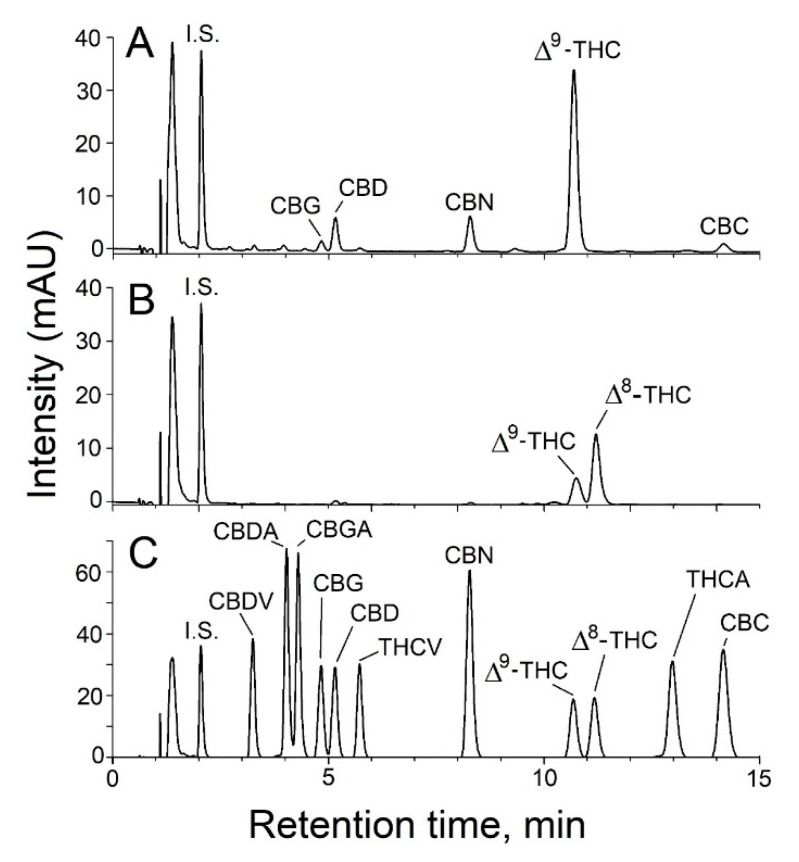
Analysis of cannabinoids in vaporizer fluids using HPLC-PDA. The cannabinoid profiles of a vaporizer fluid in which Δ^9^-THC is the most abundant cannabinoid (**A**) and one in which Δ^8^-THC is the more abundant than Δ^9^-THC (**B**) are shown. Chromatography of the cannabinoid standards is shown in panel (**C**). The internal standard, norgestrel, is indicated by I.S.

Targeted analysis of vaporizer fluids included the following analytes: 11 natural cannabinoids, five mycotoxins, VEA, the fungicide myclobutanil and the pesticide synergist, PBO. The analysis of the cannabinoid profiles in vaporizer fluids using HPLC-PDA is shown in Figure 3.

The cannabinoid profile observed in Figure 3A is typical of a cannabis oil vaporizer fluid, with Δ^9^-THC being the most abundant cannabinoid and lesser amounts of CBD, CBN, CBG, and CBC present. In contrast, the cannabinoid profile from the analysis of a different vaporizer fluid shows Δ^8^-THC rather than Δ^9^-THC as the most abundant cannabinoid, and only trace levels of other cannabinoids were present. The presence of the Δ^8^-THC isomer, which was initially identified during untargeted screening using both GC-MS and LC-HRMS/MS (Figure 2B), was confirmed in the quantitative cannabinoid analysis. The Δ^8^-THC isomer is rarely observed in common cannabis strains. While we have not previously observed significant Δ^8^-THC in medical marijuana products, we have observed significant levels of Δ^8^-THC in a subset of illicit cannabis vaporizer cartridges.

The cannabinoid levels of the vaporizer fluids are shown in Table 1. Two of the samples (4 and 16) contained nicotine and did not contain cannabinoids; the others contained cannabinoids. The levels of total cannabinoids, and notably Δ^9^-THC levels, in these illicit vaporizer oils are considerably lower than we normally observe in medical marijuana cannabis oils. Given the reduced cannabinoid content of the illicit fluids in comparison with those of high-purity cannabis oils, other substances must make up the mass difference of the oils. Since we observed what appeared to be very high levels of VEA in fluids during our untargeted screening, it was clear that a quantitative assay for VEA was necessary, and one was therefore developed (Figure S1). VEA was found to be a major and, in some cases, the most abundant compound present in the fluid. VEA was found in 23 of the 36 cannabinoid-containing cartridges (64%) ranging from 16 to 57% by mass.

MCT oil was also identified as a significant additive in many of the vaporizer fluids. MCT oil is a mixture of triglycerides, the relative amounts of which vary dependent on the source and the processing of the oil. Since analytical standards for each of the individual components of MCT are not available, we estimated MCT content by comparison of the sum of the peak areas for the [M+NH_4_]^+^ ions of the four major components from the analysis of the unknown samples in comparison with those from an identical analysis of commercial MCT. Using this approach, we estimated MCT concentrations to be in the range of 3 to 24% by mass in the 15 fluids in which MCT was detected.

### 3.3. Pesticides and Mycotoxins

Pesticide residues were also detected in vaporizer fluids. Since the fungicide myclobutanil and the pesticide synergist PBO are often detected in cannabis [14], we performed quantitative analysis for these compounds. Myclobutanil and PBO were detected in numerous samples (Table 2). Pesticide screening using the UHPLC-Q-Orbitrap-MS system identified additional pesticide residues in the vaporizer fluids. In addition to myclobutanil, several other fungicides were detected >1 µg/g: trifloxystrobin, metalaxyl, tebuconazole, cyprodinil, diphenylamine, and methyl-kresoxim. The pyrethroid insecticides trans-permethrin, cis-phenothrin, bifenthrin, and fenpropathrin were detected in some samples, as were the acaricides bifenazate and etoxazole. The mycotoxin panel required for medical marijuana product analysis in NYS includes aflatoxins B1, B2, G1, G2, and ochratoxin A. No significant mycotoxin contamination of any of the vaporizer fluids analyzed in this study was observed.

### 3.4. Analysis of Commercial Diluents and Thickeners

**Figure 4:**
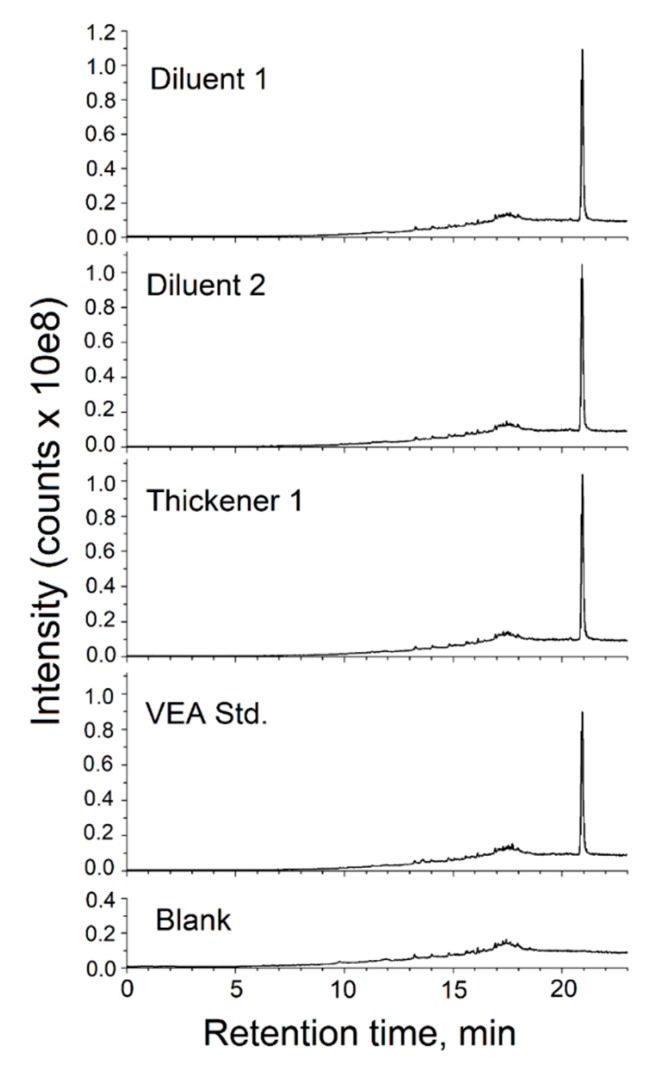
Analysis of commercial cannabis oil additives using LC-HRMS/MS. The TIC chromatograms from the analysis of diluents 1 and 2 and thickener 1 are shown together with that of the VEA standard recorded on the LC-tripleTOF system under the identical analytical conditions.

Six commercial products that were marketed as cannabis oil diluents or thickeners were obtained by the NYSDOH for analysis. These products were touted as being natural and free of vegetable glycerin and propylene glycol. The products were purchased via the internet and are referred to as diluents 1, 2 and 3 and thickeners 1, 2 and 3. These products were analyzed using NMR, GC-MS, and LC-HRMS/MS. Diluents 1 and 2 and thickener 1 were found to be essentially pure VEA (Figure 4). While LC-HRMS/MS and GC-MS showed no other component(s) in these three products, one-dimensional (1D) ^1^H and 1D ^13^C NMR indicated the presence of additional unidentified component(s) at the level of about 1% in thickener 1.

**Figure 5:**
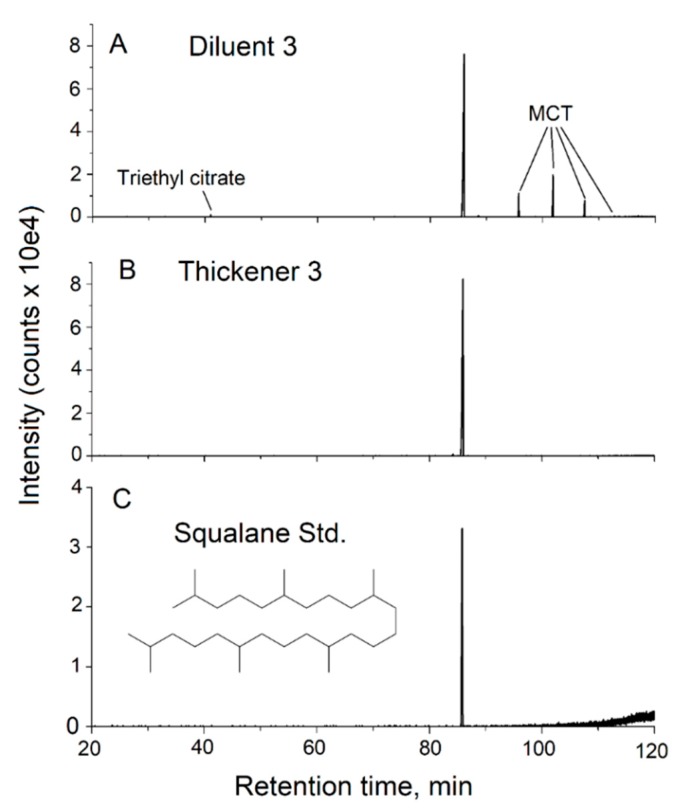
Analysis of cannabis oil additives using GC-MS. The TIC chromatograms from the analysis of diluent 3 and thickener 3 using the long GC-MS program for untargeted analysis are shown together with that of a squalene standard. Peaks representing additional components identified in diluent 3, triethyl citrate, and MCT are denoted.

The analysis of diluent 3 and thickener 3 using GC-MS is shown in Figure 5. Both show a major peak at 86 min in the long untargeted GC-MS program. Analysis of the mass spectra recorded for these components matched the NIST library spectrum for squalane (Figure S4). Analysis of a squalane standard confirmed retention times and mass spectra. Whereas thickener 3 appears to be essentially pure squalane, diluent 3 appears to be a mixture of components. The component of greatest spectral intensity was identified by the NMR analysis as squalane. The 1D ^1^H, 1D ^13^C, and two-dimensional (2D) ^1^H-^13^C heteronuclear single quantum coherence NMR spectral peaks from this component correspond quite precisely with those of the squalane standard. The identifications of MCT and triethyl citrate were verified by 1D ^1^H and 1D ^13^C NMR tested against available reference materials, commercial Miglyol MCT oil and a triethyl citrate analytical standard. The Miglyol MCT oil was verified to have a mixture of C_8_ and C_10_ chains in a 60/40 ratio with little C_12_ chain present. The relative intensities of resonances from each of the three components yielded the relative molar concentration estimates of 1.0:0.4:0.06 for squalene, MCT and triethyl citrate, respectively. GC-MS analysis was consistent with diluent 3 being a mixture of squalene, MCT and triethyl citrate (Figure S5). 1D ^1^H and 1D ^13^C NMR and GC-MS analyses in comparison with those of an analytical standard indicate the base component of thickener 2 is α-bisabolol (Figure S6). Based on a match to the NIST library mass spectrum and a comparison between calculated 1D ^1^H and 1D ^13^C NMR spectra, a minor component of this product appeared to be the terpenoid alcohol, isophytol (Figure S6). This finding was confirmed upon procurement and analysis of an isophytol standard. A summary of our analyses of the diluents and thickeners is presented in Table 3. 

## 4. Discussion

### 4.1. Characterization of Illicit Cannabis Vaporizer Fluids

After four years of developing and validating certified methods for the analysis of medical marijuana products, and then applying them to the analysis of thousands of samples in the highly regulated NYS Medical Marijuana Program (NYSMMP), we observed a sharp contrast with the results we obtained for analysis of the illicit “street” products associated with the recent EVALI outbreak with what we observe for the NYSMMP products. The illicit vaporizer fluids can be characterized as lower in cannabinoid content, sometimes with an unusual ratio of the Δ^9^- and Δ^8^-THC isomers, often containing pesticide residues and as being heavily cut with diluents, most notably with VEA.

Typical vaporized fluids in the NYSMMP are excipient-free and contain 80 to 90% total cannabinoid content. The illicit vaporizers fluids are usually <50% cannabinoids with as much as 58% VEA. Some of the fluids we analyzed showed an unusually high content of the Δ^8^-THC isomer. The Δ^8^-THC to Δ^9^-THC ratios for some of the fluids analyzed far exceeded those of known natural cannabis strains. Several possibilities exist for the observed enrichment of Δ^8^-THC. The Δ^8^-THC isomer has been legal and unregulated in some states. A market for the less-potent Δ^8^-THC isomer may have arisen due to legal loopholes. There is still no clear explanation for the presence in the fluids of what is usually a rare cannabinoid. One possibility is that a chromatographic, distillation, or selective extraction process was used to separate the two isomers. A second possibility is the accidental or intentional conversion of Δ^9^-THC to Δ^8^-THC during a high-temperature process, such as decarboxylation of THCA or vacuum distillation. A third possibility is that the Δ^8^-THC isomer is formed during a semi-synthetic production of THC, perhaps with CBD as a starting material [15]. Regardless of its origin, this distinctively high content of Δ^8^-THC is a characteristic feature of a subset of illicit vaporizer fluids. 

The pesticide contamination we observed may be associated with outdoor growing that is either illegal or poorly regulated. The most commonly detected pesticides were myclobutanil and other fungicides; however, pyrethroid insecticides and acaricides were also detected. The fact that pesticide contamination appears intermittent and random suggests that the cannabis oil in these case-associated products is not from a common source. It would also suggest that EVALI is not due to exposure to a single pesticide, since there was no consistent, high-level pesticide contamination detectable in the fluids. In any case, the pesticide contamination observed in several samples would not be permitted in most legal medical marijuana or adult-use programs.

### 4.2. VEA and Other Diluents and Thickeners

Initially, we had no explanation for our unusual finding of VEA in vaporizer fluids. When we began to detect very high VEA levels repeatedly in case-associated vaporizer fluids amid the outbreak of EVALI, we shared these findings on VEA with public health officials of the U.S. CDC, the U.S. FDA and those in numerous states via conference calls on the outbreak and via e-mail on August 19, 2019. On September 5, 2019, the NYSDOH announced an update on its investigation into vaping-associated pulmonary illness, warning against the use of black-market vaping products and indicated that VEA was a key focus of the investigation [16]. Since the alert by the NYSDOH, the U.S. FDA [17] and the U.S. states of Utah [18] and Minnesota [19] have detected VEA in EVALI case-associated vaporized fluids. VEA is a pro-vitamin form of vitamin E (α-tocopherol). In its pure form, VEA is a clear to slightly yellow, very viscous oil. It is readily available from several suppliers at low cost. Our investigations soon revealed a significant internet lore and reports of VEA being used as vaporizer fluid diluent in the illicit market [20,21,22].

It appears that VEA was recently recognized as an ideal diluent for illicit cannabis vaporizer fluids. To maximize profits and extend supply, cutting the product with cheaper material that is undetectable to the consumer has long been a practice in the illegal market for drugs such as heroin and cocaine. For cutting cannabis vaporizer fluids, diluents such as glycerol, propylene glycol, and MCT do not provide enough viscosity, such that the savvy consumer knows that the product is not pure cannabis oil. VEA-based diluents were touted as tasteless and odorless thickeners that are miscible with cannabis oil in all proportions and produce a desirable cloud on vaping. Upon visual inspection, it is nearly impossible to distinguish between a cartridge containing high-grade cannabis oil and one heavily cut with VEA. This new generation of VEA-based vaporizer diluents came to the market in 2018, but sales of these new products greatly increased in the Spring of 2019, which just preceded the onset of the nationwide outbreak of EVALI [20]. In some cases, we observed both VEA and MCT in the same vaporizer fluid. It is unclear whether the cartridges containing both VEA and MCT were originally formulated with both diluents, or originally contained MCT and were diluted (“cut”) with VEA or vice versa.

Since the firms marketing cannabis oil diluents and thickeners do not list their ingredients on the label or on their website, NYSDOH purchased six diluent and thickener products for analysis. Three of the diluents/thickeners were found to be essentially pure VEA. Two others were found to be primarily squalane, a semi-synthetic compound derived from the natural product, squalene. The sesquiterpene alcohol, α-bisabolol, was the major ingredient in the sixth product. It appears that none of these products or components have been studied regarding their safety in their intended or implied use, which is as diluents for vaporizer fluids. The vaping process involves aerosolizing fluids at 250 to 350 °C, and then inhaling the aerosol deeply into the lungs. While companies may be marketing pharmaceutical-grade reagents that are approved for other purposes such as ingestion, their safety in this vaping scenario is unknown.

### 4.3. Vaping and Pulmonary Disease

Beginning in the Spring of 2019, there was a sharp increase in EVALI, with some cases showing clinical presentation consistent with lipoid pneumonia [3,23,24]. Other reports of limited case studies suggest that the pathology of EVALI is distinct from lipoid pneumonia, describing the pathology as chemical pneumonitis [25] or in some cases organizing pneumonia [26]. While investigation into the cause(s) of EVALI is ongoing, there is currently strong evidence compiled by the FDA and the CDC implicating the use cannabis liquids in EVALI. Among the first 10 NYS cases of EVALI who provided product usage histories during case interviews, all reported use of cannabinoid-containing vaping products, and the majority (80%) reported combined use of cannabinoid- and nicotine-containing vaping products. There is extensive literature on nicotine-based vaping products and the generation of potential toxicants including free radicals produced from the pyrolysis of additives and flavorings [27,28,29,30,31]. In vivo and in vitro studies have studies have identified toxic byproducts and pathologic outcomes, and studies have been conducted to determine whether vaping nicotine products is safer than smoking. In the two nicotine-containing products we analyzed, we detected no unusual compounds that appeared to be of concern. However, far less is known of the toxicology of the less volatile vaping oils and oil-based cannabinoid vaping products. Our studies were focused on semi-volatile organic components and contaminants in these products as possible toxicants in EVALI.

Lipoid pneumonia was first described in 1925 [32]. It has been established that inhaling various oils and high-molecular-mass lipophilic compounds causes this condition. Repeated or extended inhalation and pulmonary exposure to substances including mineral oil, squalene, Vaseline, olive oil, castor oil, omega-3-acid esters, rendered animal fat, and amiodarone are known to cause lipoid pneumonia [33,34,35,36,37,38,39]. Given that VEA is also a high-molecular-mass, semi-volatile and lipophilic oil with similar chemical and physical properties (log*P*_oct/water_ >6 and boiling point >200 °C at 760 mmHg) as compounds that are known cause lipoid pneumonia, it is possible that VEA is causative or contributes to the development of EVALI and lipoid pneumonia. The high temperature of the aerosols in vaping may alter the pathology caused by vaping in comparison with what is observed with classic lipoid pneumonia.

Whether VEA causes classic lipoid pneumonia is unknown; however, aerosolized droplets of VEA could irritate the lung mucosa and bronchi, leading to chronic hypoxia [40]. VEA carries warnings regarding inhalation. If VEA is inhaled, it is recommended that the victim should be immediately removed from the contaminated area, and if symptoms such as wheezing, coughing, shortness of breath, or burning in the mouth, throat or chest occur, hospitalization may be necessary [41]. VEA is also unstable at high temperatures. Similar to other tocopherols, VEA undergoes pyrolysis at 300 °C [42], a temperature that is relevant to the vaping process [43]. At elevated temperatures, vitamin E oxidizes to highly reactive α-tocopherylquinone [44,45]. Our initial experiments indicate that numerous compounds including duroquinone, a redox-active para-quinone [46], are produced during the vaping of VEA. It is possible that, in the vaping process, VEA is the precursor to cytotoxic derivatives that contribute to lung injury.

Squalane, prepared by hydrogenation of the lipoid-pneumonia-causing squalene [47], would likewise appear to be a potentially dangerous compound in the context of vaping. Squalane is the base component of some of the next generation of cannabis oil diluents we obtained. Squalane is a fully saturated, branched-chain hydrocarbon with physical and chemical properties comparable to those of mineral oil. The physical process of vaping would appear to be a very effective process for delivering substances such as VEA and squalane to the lung epithelium, with unknown consequences. There is little scientific knowledge of the long-term health effects of vaping any material, and the cannabis-oil vaporizer diluent industry appears to be operating with minimal oversight. Product development appears to be driven by the low cost of base ingredients, taste, viscosity, miscibility with cannabis oil, and appearance of the final product to the customer on the street; however, there appears to have been no testing for or evaluation of the potential toxic effects of these products in the vaping process. There is no evidence that vaping large amounts of compounds such as VEA, squalane, or other chemicals present in diluent products on the market is safe.

## 5. Conclusions

The number of confirmed cases of EVALI has risen steadily. As of December 12, 2019, the CDC reports that there have been 2409 hospitalized cases of EVALI from all 50 U.S. states, the District of Columbia, the U.S. territories of Puerto Rico, and the U.S. Virgin Islands, resulting in 52 deaths in 26 U.S. states and the District of Columbia. As of December 10, 2019, NYSDOH has received reports of 205 EVALI cases from all regions of the state, including two deaths. Our analysis of vaporizer fluids continues. As of December 12, 2019, the Wadsworth Center has analyzed 206 vaporizer fluids from 61 NYS EVALI cases. Of these, 147 contained THC, and 59 contained nicotine. Of the 147 THC-containing fluids, 101 (69%) contained VEA. There is additional evidence of a strong association of VEA with EVALI. In the initial analyses of bronchoalveolar lavage fluids from EVALI patients, 28 of 28 fluids contained vitamin E acetate [48]. Extensive in vitro and in vivo studies will be required to establish a causative role of VEA and/or other agents in the etiology of EVALI.

## Figures and Tables

**Table 1:** Nicotine, cannabinoids, and additives in vaporizer fluids samples.

Sample	Nicotine	CBG	CBD	THCV	CBN	Δ^9^-THC	Δ^8^-THC	THCA	CBC	VEA (%)	MCT
1	-	2.13	0.91	<0.884 *	<0.884	34.4	<0.884	<1.58	<0.884	38.7	-
2	-	1.56	<1.43	<1.43	<1.43	45.1	<1.43	<2.38	<1.43	33.5	-
3	-	2.45	3.53	<1.17	<1.17	60.8	<1.17	<1.96	<1.17	<1.0	+
4	+	<0.965	<0.965	<0.965	<0.965	<0.965	<0.965	<1.61	<0.965	<1.0	-
5	-	1.61	<1.05	<1.05	1.05	41.6	<1.05	<1.75	<1.05	31.1	-
6	-	<1.15	9.46	<1.15	<1.15	42.8	<1.15	<1.92	<1.15	24.8	-
7	-	<2.72	<2.72	<2.72	<2.72	25.6	<2.72	<4.54	<2.72	33.4	+
8	-	<1.19	8.34	<1.19	<1.19	44.9	<1.19	<1.98	<1.19	19.2	-
9	-	<1.31	1.37	<1.31	<1.31	25.6	<1.31	<2.17	<1.31	44.6	-
10	-	<1.13	<1.13	<1.13	<1.13	11.6	26.7	1.88	<1.13	20.9	-
11	-	<1.38	<1.38	<1.38	<1.38	10.8	24.7	<2.29	<1.38	21.3	-
12	-	1.54	13.30	<1.25	1.59	14.7	<1.25	<2.08	<1.25	33.9	-
13	-	1.40	<1.18	<1.18	<1.18	19.7	<1.18	2.0	<1.18	16.4	-
14	-	<1.16	<1.16	<1.16	<1.16	30.1	<1.16	<1.92	<1.16	19.2	+
15	-	2.91	<0.944	<0.944	<0.944	63.2	<0.944	<1.57	1.20	<1.0	+
16	+	<1.50	<1.50	<1.50	<1.50	<1.50	<1.50	<2.50	<1.50	<1.0	-
17	-	1.49	<1.17	<1.17	<1.17	39.2	<1.17	<1.95	<1.17	30.4	-
18	-	<1.18	<1.18	<1.18	4.80	3.11	47.1	9.1	<1.18	<1.0	+
19	-	<1.21	<1.21	<1.21	<1.21	23.2	<1.21	2.1	<1.21	47.4	-
20	-	1.65	<1.34	<1.34	<1.34	33.3	<1.34	<2.24	<1.34	38.6	-
21	-	1.61	<1.14	<1.14	1.37	23.2	<1.14	<1.90	<1.14	39.6	-
22	-	<1.17	<1.17	<1.17	1.67	18.0	11.3	4.0	<1.17	57.6	-
23	-	<1.16	<1.16	<1.16	1.52	11.8	12.3	4.1	<1.16	51.5	-
24	-	2.81	<1.15	<1.15	4.21	32.1	<1.15	4.3	<1.15	<1.0	+
25	-	<1.21	<1.21	<1.21	1.90	32.7	<1.21	<2.01	<1.21	39.8	-
26	-	5.31	<1.14	<1.14	5.11	32.9	<1.14	3.6	<1.14	<1.0	+
27	-	2.15	5.72	<1.14	2.84	47.2	<1.14	<1.91	<1.14	<1.0	-
28	-	2.38	<1.13	<1.13	4.24	40.4	<1.13	<1.88	<1.13	<1.0	+
29	-	3.54	<1.58	<1.58	4.62	20.6	<1.58	7.7	<1.58	<1.0	+
30	-	4.69	<1.14	<1.14	4.40	28.3	<1.14	3.1	<1.14	<1.0	+
31	-	2.65	<1.14	<1.14	3.55	66.3	<1.14	<1.89	<1.14	<1.0	+
32	-	<1.22	<1.22	<1.22	4.28	4.80	38.1	4.4	<1.22	<1.0	+
33	-	2.14	<1.12	<1.12	6.86	34.5	<1.12	<1.86	<1.12	<1.0	+
34	-	2.23	<1.26	2.29	4.03	54.7	<1.26	<2.11	<1.26	<1.0	+
35	-	<1.16	<1.16	<1.16	<1.16	29.1	<1.16	<1.93	<1.16	51.2	-
36	-	<1.10	<1.10	<1.10	1.65	12.1	12.9	3.9	<1.10	43.5	-
37	-	<1.19	<1.19	<1.19	1.59	16.8	11.7	4.2	<1.19	38.4	-
38	-	1.95	<1.29	<1.29	1.70	27.1	<1.29	<2.15	<1.29	57.1	-

* Less-than (<) designates below the lower limit of quantitation that is indicated.

**Table 2:** Synthetic cannabinoids, synthetic opioids, mycotoxins, and pesticides in vaporizer fluids samples.

Sample	Screening for Synthetics		Mycotoxins (ng/g)	Myclobutanil (µg/g)	PBO (µg/g)	Other Pesticides Detected (>1 µg/g)
	Cannabinoids	Opioids				
1	neg *	neg	<4.8 **	1.97	1.38	bifenazate, trans-permethrin, boscalid, bifentrin
2	neg	neg	<4.8	1.64	0.84	bifenazate, diphenylamine, imidacloprid, cypermethrin, bifenthrin, fenpropathrin
3	neg	neg	<4.8	<0.25	24.1	trifloxystrobin, etoxazole, bifenthrin
4	neg	neg	<4.8	<0.25	<0.25	N.D. ***
5	neg	neg	<4.8	8.64	<0.25	N.D.
6	neg	neg	<4.8	0.42	<0.25	metalaxyl, etoxazole
7	neg	neg	<4.8	1.22	2.52	cis-phenothrin
8	neg	neg	<4.8	3.04	<0.25	metalaxyl, etoxazole
9	neg	neg	<4.8	<0.25	<0.25	trifloxystrobin
10	neg	neg	<4.8	<0.25	<0.25	N.D.
11	neg	neg	<4.8	<0.25	<0.25	N.D.
12	neg	neg	<4.8	<0.25	<0.25	N.D.
13	neg	neg	<4.8	<0.25	<0.25	imidacloprid
14	neg	neg	<4.8	0.28	5.74	bifenthrin, boscalid
15	neg	neg	<4.8	0.51	7.5	tebuconazole, bifenazate
16	neg	neg	<4.8	<0.25	<0.25	N.D.
17	neg	neg	<4.8	0.51	0.53	etoxazole, bifenthrin
18	neg	neg	<4.8	2.70	<0.25	bifenthrin
19	neg	neg	<4.8	<0.25	2.72	N.D.
20	neg	neg	<4.8	5.62	0.54	N.D.
21	neg	neg	<4.8	1.02	1.54	bifenazate
22	neg	neg	<4.8	3.53	<0.25	cis-phenothrin
23	neg	neg	<4.8	<0.25	<0.25	cis-phenothrin
24	neg	neg	<4.8	2.12	1.80	bifenthrin, trifloxystrobin, trans-permethrin, bifenazate
25	neg	neg	<4.8	6.51	10.8	trifloxystrobin, pyrimethanil, methyl-kresoxim, trans-permethrin
26	neg	neg	<4.8	<0.25	<0.25	N.D.
27	neg	neg	<4.8	4.97	11.20	bifenazate, bifentrin, trans-permethrin
28	neg	neg	<4.8	<0.25	<0.25	trifloxystrobin, bifenazate
29	neg	neg	<4.8	1.99	1.40	N.D.
30	neg	neg	<4.8	<0.25	<0.25	bifenazate, diphenylamine, metalaxy, tebuconazole, cyprodinil, propamocarb, trifloxystrobin
31	neg	neg	<4.8	5.11	3.81	bifenazate
32	neg	neg	<4.8	28.30	<0.25	N.D.
33	neg	neg	<4.8	1.96	2.32	trifloxystrobin, bifenazate, bifenthrin
34	neg	neg	<4.8	<0.25	<0.25	cyprodinil, buprofezin
35	neg	neg	<4.8	2.14	2.57	N.D.
36	neg	neg	<4.8	1.05	1.05	imidacloprid
37	neg	neg	<4.8	<0.25	<0.25	N.D.
38	neg	neg	<4.8	<0.25	I.M. ****	bifenazate

* neg indicates the screen was negative (analytes < 0.05% by mass). ** Less-than (<) designates below the lower limit of quantitation. *** N.D., not detected. **** I.M., insufficient material to test.

**Table 3:** Composition of six commercial vaporizer fluid diluents and thickeners.

Product	Components
Diluent 1	VEA
Diluent 2	VEA
Diluent 3	Squalane, MCT oil, triethyl citrate (minor)
Thickener 1	VEA
Thickener 2	α-Bisabolol, isophytol (minor)
Thickener 3	Squalane

## Supplementary Materials

The following are available online at https://www.mdpi.com/2305-6304/8/1/8/s1, Figure S1: Analysis of VEA using GC-MS with selected-ion monitoring, Figure S2: Mass spectral analysis of VEA in a vaporizer fluid, Table S1: Accurate mass analysis of MCT in a vaporizer fluid, Figure S3: Analysis of commercial MCT using LC-HRMS/MS, Figure S4: Mass spectral analysis of squalane in a vaporizer fluid diluent, Figure S5: Mass spectral analysis of triethyl citrate in a vaporizer fluid diluent, Figure S6: Analysis of a vaporizer fluid thickener using GC-MS.
