# Supplementary material for: Analysis of Cannabinoid-Containing Fluids in Illicit Vaping Cartridges Recovered from Pulmonary Injury Patients: Identification of Vitamin E Acetate as a Major Diluent
Source: Toxics. 2020 Jan 24;8(1):8. doi: 10.3390/toxics8010008 (PMC7151740; doi:10.3390/toxics8010008)
Supplement: Supplementary file 1 [file toxics-08-00008-s001.pdf]

Toxics **2020**, *8*, 8; doi:10.3390/toxics8010008

Supplementary Materials: Analysis of Cannabinoid-Containing Fluids in Illicit Vaping Cartridges Recovered from Pulmonary Injury Patients: Identification of Vitamin E Acetate as a Major Diluent

Bryan Duffy, Lingyun Li, Shijun Lu, Lorie Durocher, Mark Dittmar, Emily Delaney-Baldwin, Deepika Panawennage, David LeMaster, Kristen Navarette and David Spink

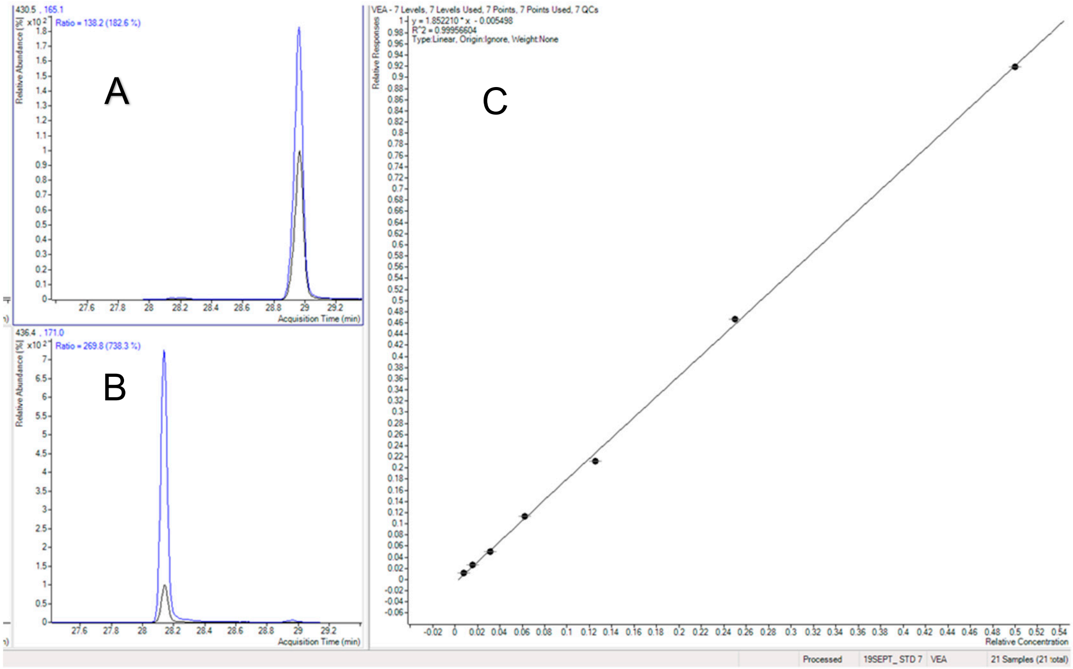

Figure S1. Analysis of VEA using GC-MS with selected-ion monitoring. In panel (**A**), selected-ion chromatograms at *m*/*z* 430 (blue) and 165 (black) for the detection of VEA, and in panel (**B**) the selected-ion chromatograms at *m*/*z* 436 (blue) and 171 (black) for the vitamin E-*d*6 internal standard are shown for calibrator 7. In panel (**C**), the calibration curve plotting the ratios of the *m*/*z* 430 peaks relative to those of the *m*/*z* 436 peaks over the range of 0.039 to 2.5 µg/mL VEA is shown, with R^2 = 0.9996 for the calibration curve.

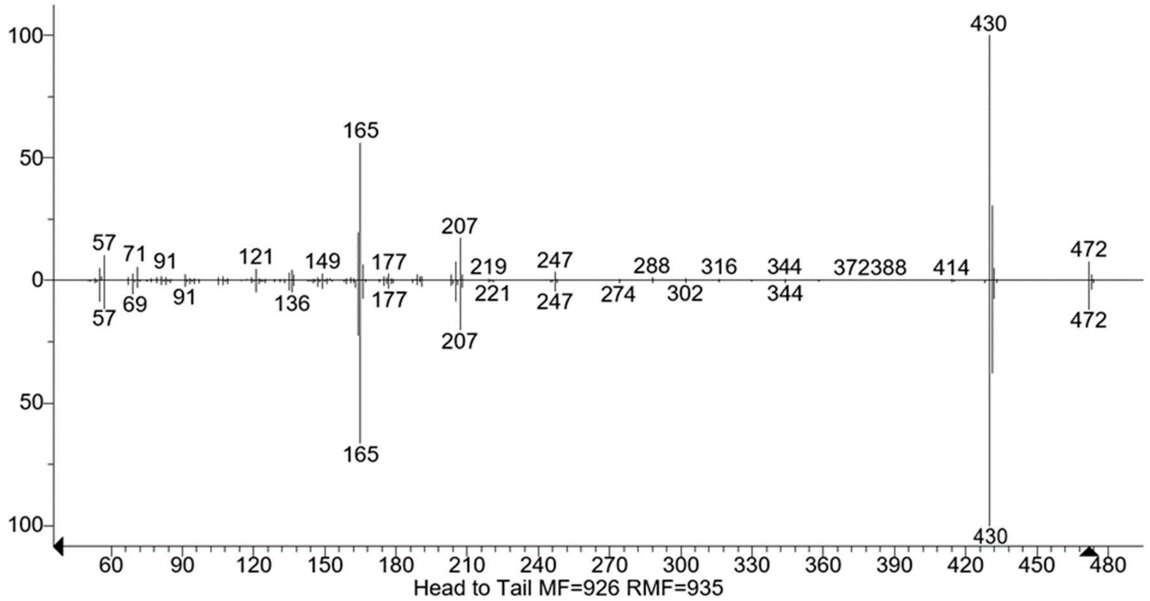

Figure S2. Head-to-tail comparison of the electron ionization mass spectrum recorded for VEA (upper) from the analysis of the cannabinoid-containing vaporizer fluid in Figure 1 with that of the NIST library mass spectrum (lower).

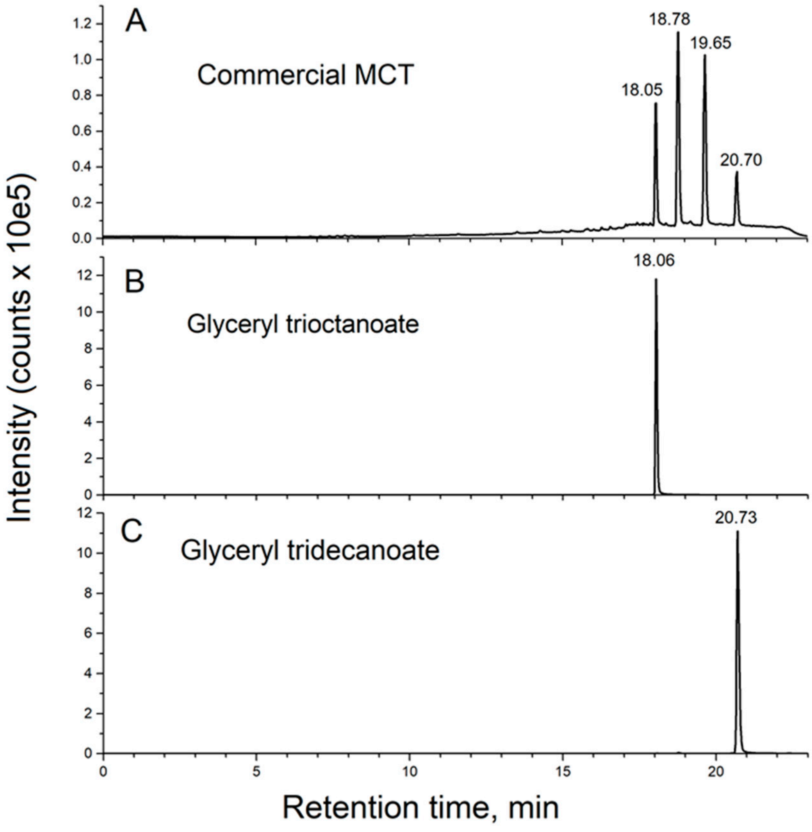

Figure S3. Analysis of commercial MCT using LC-HRMS/MS. Analysis of the commercial MCT product, Miglyol (**A**), together with a glycerol trioctanoate standard (**B**) and a glycerol tridecanoate (**C**) is shown. For each component, accompanying high-resolution MS and MS/MS data are consistent with the assigned structures in Table S1.

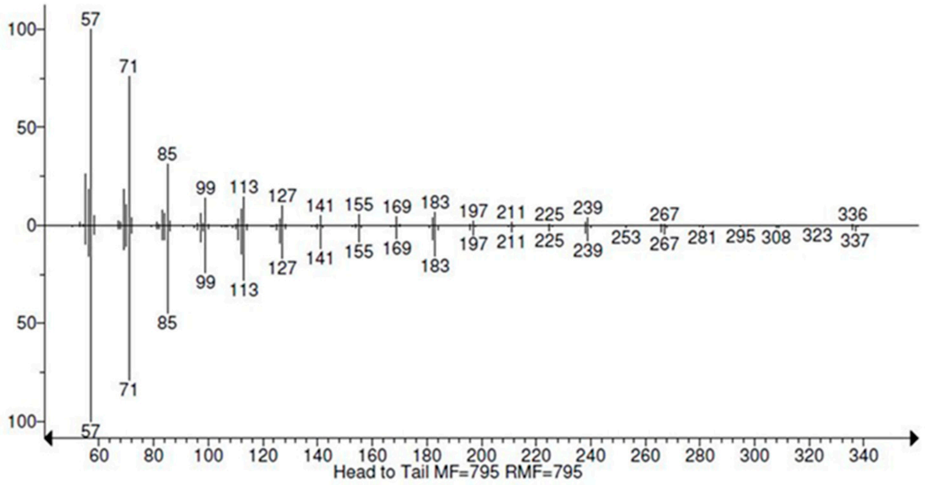

Figure S4. Mass spectral analysis of squalane in a vaporizer fluid diluent. The upper elctron-ionization mass spectrum from the GC-MS analysis of thickener 3 is shown in comparison with the NIST library spectrum of squalane.

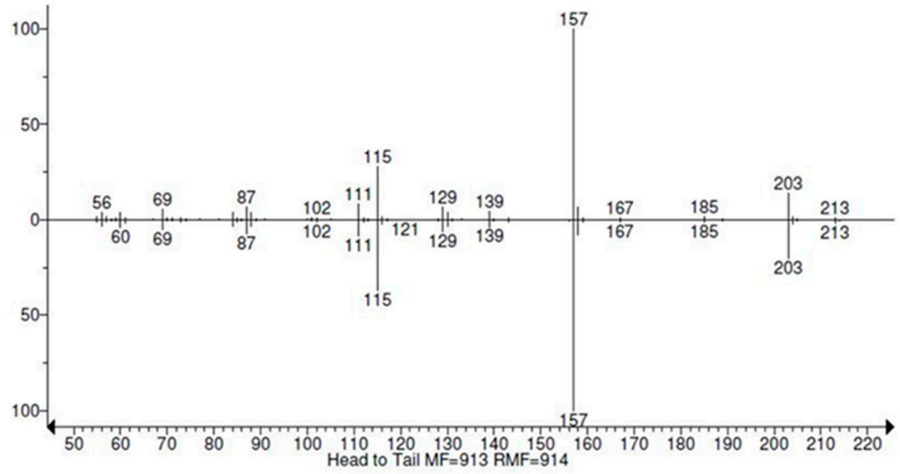

Figure S5. Mass spectral analysis of triethyl citrate in a vaporizer fluid diluent. The upper electron-ionization mass spectrum of the minor component from the GC-MS analysis of thickener 3 is shown in comparison with the NIST library spectrum of triethyl citrate.

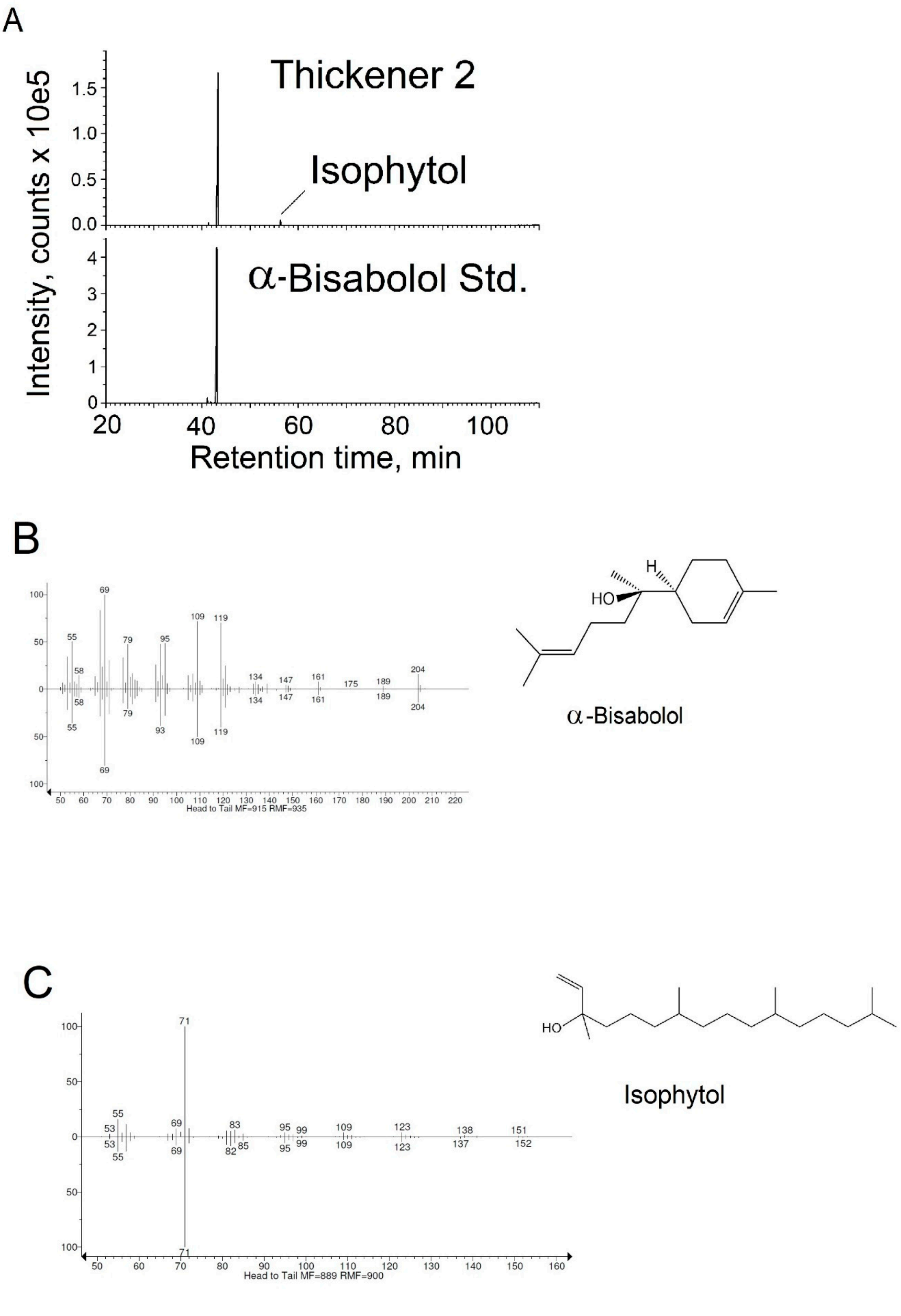

Figure S6. Analysis of thickener 2 using GC-MS. Shown in panel (**A**) are the TIC chromatograms from the analysis of thickener 2 and the α-bisabolol standard. In panel (**B**), the mass spectrum recorded for thickener 2 is shown in comparison with the NIST spectrum for α-bisabolol. In panel (**C**), the mass spectrum of the minor component of thickener 2 is compared with the NIST library spectrum of isophytol.

Table S1. Accurate mass measurements of MCT components observed from analysis of a THC-containing vaporizer fluid. The accurate masses of the putative $[M+NH_4]^+$ ions of the components denoted as MCT in Figure 2 are presented sequentially as 1) through 4) below together with their proposed structures.

**Proposed structure**	**Accurate Mass of the $[M+NH_4]^+$ Ion**		
	**Observed**	**Theoretical**	**Δ (ppm)**
1) Glyceryl trioctanoate ($C_{27}H_{50}O_6$)	488.392	488.3946	−4.85
2) Glyceryl dioctanoate-decanoate ($C_{29}H_{50}O_6$)	516.425	516.426	−0.91
3) Glyceryl didecanoate-octanoate ($C_{31}H_{58}O_6$)	544.4571	544.4572	−0.13
4) Glyceryl tridecanoate ($C_{33}H_{62}O_6$)	572.4889	572.4885	0.75
